# Predictors to parental knowledge about childhood immunisation/EPI vaccines in two health districts in Cameroon prior to the introduction of 13-valent Pneumococcal Conjugate Vaccines (PCV-13)

**DOI:** 10.11604/pamj.2014.17.187.1911

**Published:** 2014-03-11

**Authors:** John Njuma Libwea, Marie Kobela, Jukka Ollgren, Irene Emah, Robert Tchio, Hanna Nohynek

**Affiliations:** 1 School of Medicines, University of Tampere, Finland; 2 National Institute of Health & Welfare, Helsinki, Finland; 3 Expanded Immunisation Programme, Yaounde, Cameroon

**Keywords:** knowledge, attitudes, parents, guardians, PCV-13, EPI, new vaccines introduction, pneumonia, Cameroon, immunisation

## Abstract

**Introduction:**

Pneumonia is vaccine-preventable, but the increasing death toll resulting from the disease in Sub-Saharan Africa is alarming. Several factors account for vaccine failing to reach every child, besides incomplete vaccine coverage. Most of these include the perceptions of parents/guardians and healthcare providers. Previous studies on the introduction of new vaccines have focused on experimental trials, coverage figures and vaccine efficacy in developed countries. Little is known on the factors which may hinder the implementation process despite the huge challenges this may encounter in developing countries. This study described the knowledge, attitude and practices (KAP) of parents/guardians on pneumonia and immunisations/EPI vaccines; identify predictive parental socio-economic/demographic characteristics that of good knowledge on pneumonia infections, routine EPI vaccines and the PCV-13. Finally, the study described health center personnel perceptions about immunisations.

**Methods:**

The WHO's immunisation coverage cluster survey design was used, involving parents/guardians (n = 205) of children aged 0-59 months and health centre personnel (n = 13) directly concerned with vaccination activities between July-September 2010 in two health districts in Yaounde, Cameroon. Descriptive statistics and multivariate logistic models were used to analyse the parental/guardian data while the health personnel data was only analysed descriptively using SPSS version 17.0.

**Results:**

Only 19% of the parents/guardians were aware of the availability of the PCV-13. Logistic modelling identified important associations between parental socio-economic/demographic factors and good knowledge on pneumonia disease burden and prevention.

**Conclusion:**

According to parents/guardians a short and clear message on the dangers of pneumonia and the need for prevention provided to parents/guardians during sensitisation/out-reach campaigns and use of social network avenues would be primordial, if the PCV-13 is to reach every child.

## Introduction

Pneumonia remains one of the most prolific and forgotten killers to children in the class of Acute Respiratory Infections (ARI) which accounts for some 6.3% of Disability Adjusted Life Years Lost (DALYs) in children under-five worldwide [1]. According to a joint WHO/UNICEF report in 2006, pneumonia is confirmed to be a mass killer of children more than AIDS, malaria and measles combined. For many countries in Sub-Saharan Africa where most of the two million yearly under-five deaths are reported, the proportion of disease burden averages 20% [2, 3].

Pneumonia, like many other communicable infections is vaccine-preventable but the increasing death toll from the disease is a call for concern. Although the development of an efficacious PCV is a major advance in public health, mean vaccination coverage figures in most Sub-Saharan countries have not followed similarly [4, 5]. This paints a vivid picture of the incomplete coverage and under-utilization of existing vaccines in many parts of the world, with most of this in resource-poor settings [6–8].

However, research on what determines utilization of infant vaccination in Cameroon just like in most countries in the sub-region has not been rigorously considered. Previous studies in different countries have revealed a number of variables that are associated with parental knowledge about childhood vaccinations [8–10]. Such determinants include demographic, socio-economic, health system and policies beside others [7, 11]. Most of the available data on new vaccines introduction in developing countries as is with the PCV-13 has mainly focused on vaccine finance and evidence of pneumonia disease burden in children under-five years [12–14]. This makes it challenging to compare the PCV-13 and new vaccine implementation determinants in low-resource settings. To improve on the access to vaccines and reduce the burden of vaccine-preventable infections generally, such information would be essential in the implementation of new and under-used vaccines interventions in developing countries.

To describe the socio-economic/demographic determinants and predictors to parental knowledge about childhood immunisation including the accessibility of the PCV-13, we conducted an exploratory cross-sectional study in a semi-urban zone in Yaounde-Cameroon, Sub-Saharan Africa. The analysis focused on the description of common socio-economic and demographic factors and the associations between parental knowledge about childhood immunisations/EPI vaccines, pneumonia infections and prevention. Our specific interest was the identification of factors that would be predictive for a wider access of the PCV-13 to the target population.

## Methods

### Study site

The study was undertaken from July 2010 to September 2010 in a densely populated semi-urban-slum zone in Yaoundé within the Biyem-Assi and Cite Verte health districts. These two health districts were chosen because they were described as “Non-Performant” based on statistics from a rapid evaluation of vaccination coverage on the first phase of the National Immunisation days against Polio and Yellow Fever of 4-9 May 2009 [15, 16]. Yaoundé is located between Latitude 3° 52’ N and Longitude 11° 31'E. It is one of the most populated cities in Cameroon with many urban-slum dwellings and a population of over 1.8 million inhabitants [17].

### Recruitment of participants

The participants consisted of parents/guardians of children aged between 0-59 months in Biyem-Assi and Cite Verte health districts of Cameroon with a population of over four hundred thousand inhabitants. This population includes 18% of children under-five years old [17]. Participants were recruited based on the World Health Organization's Standard Reference Cluster Sampling Technique [18]. Recruitment was open to parents/guardians who showed proof of being a parent/guardian of at least one child, residing in the selected locality for at least six months, and whose child is eligible for routine vaccines scheme. Informed consent was sorted by verbatim from participants before their enrolment. Participants were able to end participation at any point or by refraining from responding to all sections of the questionnaire. The research protocol was reviewed and approved by the Cameroon National Ethics committee and the University of Tampere Medical School IRB.

### Study design and sample size estimation

The research design used is Cross-sectional Cluster survey. Primary data was obtained using structured questionnaires. The sample size estimation is in accordance with the World Health Organization's guidelines for estimating immunisation coverage in an area. A desired precision of ±10%, a design effect of 2 and a confidence level of 95% have been assumed [18]. The two health districts were divided into 25 clusters before randomly sampled.

### Collection of baseline data

#### Measurements and description of variables

Details of the data collection have been reported elsewhere [19]. In summary, participants were questioned about their demographic and socio-economic information, knowledge/attitudes and perceptions about childhood vaccinations, pneumonia disease burden and its prevention. Prior to the analyses, the most important baseline characteristics of the study were defined and some re-categorized where necessary. We identify independent socioeconomic/demographic determinants which are positively associated to parental knowledge on pneumonia disease burden and prevention, while considering their knowledge of existing EPI vaccines.

#### Statistical analysis

The data was entered, transferred to and analysed using Microsoft Access 2007, Microsoft Excel 2007 and SPSS 17.0 version respectively. In the clustered sampling analyses, three models were involved i.e. bivariate analyses using p ≥ 0.20 as elimination point, multivariate analyses where the stepwise backward logistic regression method was implicated and finally adjusted models. The variables of age, religion and parity were used for adjustment because we described them as potential confounders when considering the relationship between the socio-economic/demographic characteristics and the outcomes in the study. The outcomes sought from the models included parental knowledge on: (i) pneumonia diseases, (ii) seriousness/consequences, (iii) causes/risk factors, (iv) prevention and (v) availability of vaccines against pneumonia infections. The gender variable was sidelined in the multivariate analyses since the proportion of female respondents was higher than the males. We have expressly reported on the results of the adjusted models but some aspects from the bivariate analyses and confounders have been commented upon. The full results have been reported elsewhere [19].

**Assumption 1:** In the multivariate analyses, a p-value of 0.157 has been blindly used for variables with either binary or polynomial categories (degree of freedoms) as this has not considerably changed the end results. Usually, in the Akaike Information Criteria (AIC), different p-values are used routinely as cut-off points for statistical significance depending on the degrees of freedom of the variables [20].

**Assumption 2:** Possible dependencies due to clustering were not taken into account in the analyses. Actually, the use of several clusters requires a used factor which takes into account the clustering or at least standard errors modified to account for dependency within people in the clusters. Since the study was exploratory rather than confirmatory, we assumed any such dependency-effects will not alter the trend of results.

The choice of using the AIC is dependent on the fact that, there is no specific reason to stick to a p-value of 0.05, or low p -values as implied by applying Bayesian Information Criteria (BIC). Using AIC has been recommended [21], and the utilisation of even higher p -values (p <0.20 or p <0.50) have been found to provide more power for the selection of predictors with relatively weak effects [22], and to provide better predictions in small data sets with a set of established candidate predictors [20].

## Results

**Figure 1:**
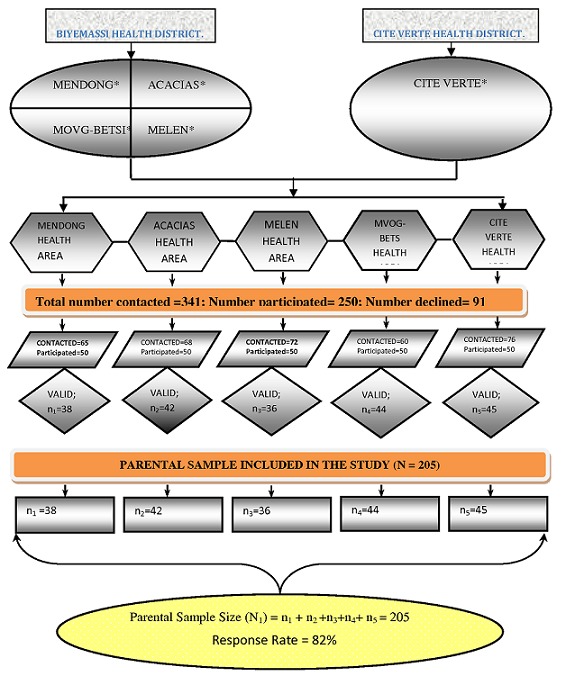
Flow chart of study participants

**Study participants:** of the 341parents/guardians contacted, 91 declined while 250 participated out from which 45 were non-respondents, resulting to the inclusion of 205 in the analysis. Figure 1: Flow chart of study participants shows the flow on the selection process. The important baseline data of the study participants are summarised in Table 1.

**Table 1:** Basic demographic and socio-economic characteristics of the study population (Baseline characteristics of the 205 participants)

Demographic variables			Socio-economic variables		
	n	%		n	%
**Gender**			**Educational level**		
Male	9	4.4	≤ Primary education	45	22.0
Female	196	95.6	Secondary/vocational education	111	54.1
**-**	-	-	≥ University education	49	23.9
**Age of respondents (in years)**			**Monthly disposal income (in Euros)**		
<25	59	28.8	1^st^ Tertile (< €76)	70	34.1
25-30	93	45.3	2^nd^ Tertile (€76-107)	73	35.6
>30	53	25.9	3^rd^ Tertile (> €107)	62	30.3
**No. of persons/household**			**Major occupation**		
≤4 person	82	40.0	Unemployed	98	47.8
Either 5 or 6 persons	67	32.7	Student	24	11.7
≥7persons	56	27.3	Employed	83	40.5
**Indigenous status**			**Membership in a social group**		
Indigene	18	8.8	Yes	95	46.3
Non-indigene	187	91.2	No	110	53.7
**Region of origin**			**Church/Religious membership**		
AD, NO, FN & foreigners	50	24.4	Christians	191	93.2
CE, SU & ES	44	21.5	Others	14	6.8
LT & SW Regions	19	9.2	-	-	-
NW & OU Regions	92	44.9	-	-	-

AD: Adamawa, NO: North, FN: Far North, CE: Centre, SU: South, ES: East, LT: Littoral, SW: Southwest, NW: Northwest, OU: West.

**Parental knowledge-attitudes about pneumonia and opinions on immunisation/PCV-13:** ninety four percent of the participating parents said they knew the types of vaccines their children had taken, while 6% had no knowledge. The current EPI vaccines which parents most often remembered that their children had taken or were due to take included: BCG, Polio, DTP (diphtheria, tetanus, and pertusis), measles and the yellow fever vaccines. This also applied to vitamin A supplements. When the respondents were questioned on what they thought could be done for the PCV-13 to effectively reach every child, 56% mentioned via an increase in sensitisation/campaigns. Relevant details are shown on Table 2 and full details have been reported elsewhere [19].

**Table 2:** Parental knowledge-attitudes and opinions about pneumonia and immunisations/PCV-13 (n= 205)

Knowledge variables			Attitude/practice & opinion variables		
	n	%		n	%
**≥1 symptoms of pneumonia**			**Child vaccination protects health**		
Answered correctly	142	69.3	Agreed	199	97.1
Don't know	63	30.7	-	-	-
**Disease burden**			**Child vaccination is against my religious belief**		
Good	39	19.1	Agreed	2	1.0
Moderate	112	54.6	-	-	-
Don't know	54	26.3	-	-	-
**≥1 Seriousness/consequences**			**Child immunisation reduces expenses on medication**		
Answered correctly	128	62.4	Agreed	171	83
Don't know	128	62.4	-	-	-
**≥1 Causes/risk factors**			**Child immunisation is against the cultural practice of my community**		
Answered correctly	135	65.9	Agreed	3	1.5
Don't know	70	34.1	-	-	-
**≥1 Prevention method**			**To take a child for vaccination is a time-wasting exercise**		
**Answered correctly**	198	96.6	Agreed	32	15.6
**Don't know**	7	3.4	-	-	-
**Availability of vaccines**			**Parental impression on health personnel**		
Yes	38	18.5	Impressive	163	79.5
No	25	12.0	Not impressive	20	9.8
Don't know	142	69.5	Others	22	10.7

### Socio-economic/demographic determinants (SED) and predictors to parental knowledge about immunisations, pneumonia infections and prevention

**Association between parental SED factors and having good knowledge of pneumonia disease burden:** membership in a social group was positively associated parental knowledge on pneumonia disease burden. Thus, parents who were members of a social group had increase odds of having “good knowledge” on pneumonia disease burden by nearly 60% (AOR = 1.57, CI = O.70-3.50) than those who were not members in a social group. There was a 30% difference between bivariate odds ratio and that of the adjusted odds ratio (Table 3).

**Table 3:** Association between socio-economic/demographic variables and correct parental knowledge on pneumonia disease burden: Odds Ratio (OR) and 95% Confidence Interval (95% CI). Model 1 = Bivariate analyses; Model 1^a^ = Multivariate analyses; Model 1^b^ = Adjusted Odds ratio analyse

	Model 1			Model 1^a^			Model 1^b^		
	OR	95%CI	P-value	AOR	95%CI	P-Value	AOR	95%	P-Value
**Gender**									
Male	2.22	0.53-9.31	0.275						
Female	1.00	Reference	0.137						
**Age in Years**									0.285
<25	0.52	0.21 – 1.28	0.153				0.52	0.16 – 1.64	0.262
25-30	0.45	0.20 – 1.02	0.057				0.46	0.17 – 1.22	0.117
>30	1.00	Reference	Reference				1.00	Reference	Reference
**Education Level**			0.783						
Low	0.75	0.27 – 2.44	0.574						
Medium	0.76	0.42 – 2.33	0.514						
High	1.00	Reference	Reference						
**Monthly disposal income**			0.993						
1^st^ Tertile	0.95	0.40 -2.27	0.950						
2^nd^ Tertile	0.99	0.42 – 2.33	0.500						
3^rd^ Tertile	1.00	Reference	Reference						
**Occupation**			0.313						
Unemployment	0.57	0.27 – 1.20	0.138						
Student	0.63	0.19 – 2.06	0.445						
Employed	1.00	Reference	Reference						
**Membership in a social group**									
Yes	1.89	0.92 – 3.81	0.081	1.89	0.92 – 3.81	0.081	1.57	0.70 – 350	0.274
No	1.00	Reference	Reference	1.00	Reference	Reference	1.00	Reference	Reference
**Religion**									
Christian	1.44	0.31 – 1.92	0.641				1.30	0.27 – 6.20	0.743
Others	1.00	Reference	Reference				1.00	Reference	Reference
**Parity**			0.921						0.742
One child	0.85	0.38 – 1.92	0.696				1.40	0.51 – 3.88	0.518
Two child	0.88	0.36 – 2.17	0.775				1.46	0.81 – 4.18	0.478
Three or more children	1.00	Reference	1.000				1.00	Reference	Reference

**Association between SED factors and having good parental knowledge on the seriousness/consequences of pneumonia infections:** educational status was associated with a decreased odd of having good knowledge on the consequences/seriousness of pneumonia infections. Therefore, the odds of parents/guardians with ≤ primary education of having good knowledge on the consequences/seriousness of pneumonia infections were 68% (AOR= 0.32, CI = 0.13 - 0.78) less than those with university backgrounds. Likewise, the odds of parents/guardians with secondary/vocational education to have good knowledge on the consequences/seriousness of pneumonia infections were 27% (AOR = 0.73, CI = 0.34 - 1.57) less than those with university education (Table 4).

**Table 4:** Association between socio-economic/demographic variables and correct parental knowledge on the seriousness/consequences of pneumonia infections

Social-economic/demographic variables	Assessment of parental knowledge on the seriousness/consequence of pneumonia infection (modelling for “don't know”)								
	Model 1			Model 1^a^			Model 1^b^		
	OR	95%CI	P-value	OR	95%CI	P-value	OR	95%CI	P-value
**Gender**									
Male	1.21	0.29 – 4.99	0.789						
Female	1.00								
**Age in years**									
<25	0.88		0.812				0.74	0.28 – 1.98	0.551
25 – 30	1.10	0.41 – 1.89	0.750				0.88	0.39 – 1.98	0.876
>30	1.00	0.55 – 2.23	0.786				1.00		
**Region of origin**									
Pool 1	0.55	0.27 – 1.11	0.301						
Pool 2	1.09	0.51 – 2.35	0.096						
Pool 3	0.70	0.26 – 1.92	0.828						
Pool 4	1.00		0.486						
**Educational Level**									
Low	0.29	0.12 – 0.69	0.013			0.013	0.32		0.028
Medium	0.67	0.32 – 1.40	0.005	0.29	0.12 – 0.69	0.005	0.73	0.13 – 0.78	0.013
High	1.00	Reference	0.286	0.67	0.32 – 1.40	0.286	1.00	0.34 – 1.57	0.726
**Monthly disposal income**									
1^st^ Tertile	0.56		0.272						
2^nd^ Tertile	0.75	0.27 – 1.14	0.108						
3^rd^ Tertile	1.00	0.37 – 1.55	0.439						
**Occupation**									
Unemployment	0.61	0.33 – 1.12	0.282						
Student	0.76	0.30 – 1.96	0.112						
Employment	1.00	Reference	0.571						
**Membership in a social group**									
Yes	1.06	0.60 – 1.87	0.843						
No	1.00	Reference							
**Religion**									
Christian	3.26	1.05 – 10.11	0.041				3.04	0.95 – 9.69	0.061
Others	1.00	Reference					1.00		
**Parity**									
One child	1.12	0.58 – 2.15	0.533				1.05	0.44	0.907
Two child	1.52	0.72 – 3.23	0.743				1.28	0.53	0.581
Three or more children	1.00	Reference	0.271				1.00		

Odds Ratio (OR) and 95% Confidence Interval (95% CI). Model 1 = Bivariate analyses; Model 1^a^ = Multivariate analyses; Model 1^b^ = Adjusted Odds ratio analyses

**Association between SED factors and having good parental knowledge on the causes/risk factors of pneumonia infections:** both lower educational status and lower income level were associated with decreased odds of good parental knowledge on the causes/risk factors of pneumonia infections. Thus, the odds of parents/guardians with lower incomes to have good knowledge on the causes/risk factors of pneumonia were 55% (CI = 0.20 - 0.99) less than those in the 3rd tertile income level. More so, the odds of parents/guardians with ≤ primary education having good knowledge on the causes/risk factors of pneumonia infections is 76% (CI = 0.09-0.64) less, than in those with university education (Table 5).

**Table 5:** Association between socio-economic/demographic variables and correct parental knowledge on the causes/risk factors for pneumonia infections

Social-economic/demographic variable	Assessment of parental knowledge on the cause/risk factors of pneumonia infection (modelling for “don't know”)								
	Model 1			Model 1^a^			Model 1^b^		
	OR	95%CI	P-value	OR	95%CI	P-value	OR	95%CI	P-value
**Gender**									
Male	0.40	0.1 – 1.53	0.179	0.28	0.07 – 1.16	0.078	0.26	0.05 – 1.21	0.086
Female	1.00	Reference							
**Age in years**									
<25	0.92	0.42 – 2.03	0.916				1.17	0.69 – 1.98	0.560
25 – 30	0.86	0.42 – 1.76	0.838				*	*	*
>30	1.00	Reference	0.677				1.00	Reference	
**Region of origin**									
Pool 1	0.69	0.34 – 1.41	0.778						
Pool 2	0.89	0.42 – 1.91	0.690						
Pool 3	0.99	0.35 – 2.89	0.765						
Pool 4	1.00		0.997						
**Educational level**									
Low	0.20	0.08 – 0.51	0.002	0.23	0.09 – 0.58	0.004	0.24	0.09 – 0.64	0.008
Medium	0.56	0.25 – 1.24	0.001	0.60	0.27 – 1.35	0.002	0.64	0.28 – 1.49	0.004
High	1.00	Reference	0.152			0.215			0.302
**Monthly disposal income**									
1^st^ Tertile	0.40	0.19 – 0.85	0.053	0.45	0.20 – 0.98	0.133	0.45	0.20 – 0.99	0.145
2^nd^ Tertile	0.56	0.31 – 1.40	0.017	0.64	0.29 – 1.41	0.045	0.63	0.28 – 1.40	0.050
3^rd^ Tertile	1.00	Reference	0.269	1.00		0.263	1.00		0.258
**Occupation**									
Unemployed	0.58	0.31 – 1.09	0.234						
Student	0.77	0.29 – 2.03	0.089						
Employed	1.00	Reference	0.593						
**Member in a social group**									
Yes	1.35	0.76 – 2.42	0.310						
No	1.00	Reference							
**Religion**									
Christian	2.03	0.68 – 6.04	0.203				0.62	0.20 – 1.96	0.148
Others	1.00	Reference					1.00		
**Parity**									
One child	1.15	0.59 – 2.24	0.474				0.89	0.56 – 1.42	0.625
Two child	1.61	0.75 – 3.48	0.676				*	*	*
Three or more children	1.00	Reference	0.226						

Odds Ratio (OR) and 95% Confidence Interval (95% CI). Model 1 = Bivariate analyses; Model 1^a^ = Multivariate analyses; Model 1^b^ = Adjusted Odds ratio analyses

**Association between SED factors and having good parental knowledge on the prevention against pneumonia infections:** in the occupational group, being a student was associated with parental knowledge on pneumonia prevention. Hence, the odds of parents/guardians in the student occupational group to have good knowledge on pneumonia prevention methods were 97% (AOR = 0.03, CI = 0.001-0.92) less, than with those employed. Though a confounder, there was a positive association between age and parental knowledge of pneumonia preventive methods. Parents in the < 25years category, had 270% odds of having good preventive knowledge than those aged above 30years old. However, there is a more than 25-fold increase in the adjusted odds ratio from that obtained in the bivariate analyses (Table 6).

**Table 6:** Association between socio-economic/demographic variables and correct parental knowledge on the prevention against pneumonia infections

Socio-economic/demographic variables	Assessment of parental knowledge on prevention against pneumonia infection (modelling for “don't know”)								
	Model 1			Model 1^a^			Model 1^b^		
	OR	95%CI	P-value	OR	95%CI	P-value	OR	95%CI	P-value
**Gender**									
Male	8	8	8						
Female	1.00	Reference							
**Age in years**									
<25	1.12	0.22 – 5.80	0.314				27.04	0.95 – 768.50	0.062
25 – 30	5.52	0.56 – 54.67	0.893				28.71	1.54 – 534.05	0.053
>30	1.00	Reference	0.144				1.00		0.024
**Region of origin**									
Pool 1	0.35	0.06 – 2.16	0.641						
Pool 2	0.96	0.08 – 10.83	0.257						
Pool 3	0.40	0.03 – 4.65	0.971						
Pool 4	1.00	Reference	0.464						
**Educational level**									
Low	3.91	0.42 – 36.39	0.154						
Medium	4.84	0.86 – 27.40	0.231						
High	1.00	Reference	0.074						
**Monthly disposal income**									
1^st^ Tertile	0.74	0.12 – 4.61	0.876						
2^nd^ Tertile	1.18	0.16 – 8.66	0.751						
3^rd^ Tertile	1.00	Reference	0.868						
**Occupation**									
Unemployment	1.19	0.16 – 8.60	0.069	1.19	0.16 – 8.60	0.069	0.38	0.04 – 4.19	0.124
Student	0.17	0.03 – 1.10	0.867	0.17	0.03 – 1.10	0.867	0.03	0.001 – 0.920	0.433
Employed	1.00	Reference	0.063	1.00	Reference	0.063			0.045
**Membership in a social group**									
Yes	1.16	0.25 – 5.31	0.851						
No	1.00	Reference							
**Religion**									
Christian	0.00	8							
Others	1.00	Reference	0.999						
**Parity**									
One child	0.30	0.03 – 2.70	0.557				0.15	0.01 – 2.55	0.234
Two child	0.39	0.03 – 4.40	0.280				0.09	0.01 – 1.45	0.189
Three or more children	1.00	Reference	0.445				1.00	Reference	0.086

Odds Ratio (OR) and 95% Confidence Interval (95% CI). Model 1 = Bivariate analyses; Model 1^a^ = Multivariate analyses; Model 1^b^ = Adjusted Odds ratio analyses

Association between parental SED factors and having good knowledge on the availability of vaccines (PCV) against pneumonia infections: there was a positive association between Region of origin and good knowledge on the availability of vaccines against pneumonia infections. Parents who originate from Pool 1 had increased odds (AOR = 3.67, CI = 1.47-9.20) of having good knowledge on the availability of pneumonia vaccines than those from Pool 4 (Table 7).

**Table 7:** Association between SED variables and positive parental knowledge on the availability of vaccines against pneumonia infections (Odds Ratio and 95 CI)

	Socio-economic/demographic variables (SED)						Assessment of parental knowledge on the availability of vaccines against pneumonia (modelling for “don't know”)											
	Model 1						Model 1^a^						Model 1^b^					
	Yes			No			Yes			No			Yes			No		
	OR	95%CI	P-value	OR	95%CI	P-value	OR	95%CI	P-value	OR	95%CI	P-value	OR	95%CI	P-value	OR	95%CI	P-value
**Gender**																		
Male	1.26	0.24 – 6.50	0.000			0.000												
Female	1.00	Reference	0.783	0.94	0.11 – 8.20	0.959												
**Age in years**																		
<25	1.04	1.39 – 2.80	0.000	0.68	0.24 – 1.92	0.001												
25 – 30	1.01	0.41 – 2.46	0.940	0.35	0.12 – 1.00	0.466							1.12	0.31 – 4.10	0.856	0.40	0.10 – 1.39	0.14
>30	1.00	Reference	1.000			0.050							0.77	0.27 – 2.22	0.627	0.25	0.07 – 0.83	0.02
**Region of origin**																		
Pool 1	3.41	1.39 – 8.36	0.000	0.40	0.11 – 1.47	0.000	3.41	1.39 – 8.36	0.000	0.40	0.11 – 1.47	0.000	3.67	1.47 – 9.20	0.006	0.48	0.13 – 1.81	0.22
Pool 2	2.05	0.75 – 5.57	0.007	0.85	0.30 – 2.40	0.168	2.05	0.75 – 5.57	0.007	0.85	0.30 – 2.40	0.168	1.96	0.72 – 5.36	0.191	0.89	0.31 – 2.55	0.82
Pool 3	1.24	0.31 – 5.02	0.160			0.764	1.24	0.31 – 5.02	0.160			0.764	1.22	0.30 – 4.97	0.786			
Pool 4	1.00	Reference	0.766	8	8	8	1.00	Reference	0.766	8	8	8				8	8	8
**Educational level**																		
Low	1.17	0.43 – 3.21	0.001	1.41	0.39 – 5.09	0.000												
Medium	0.76	0.32 – 1.85	0.757	1.21	0.40 – 3.61	0.603												
High	1.00	Reference	0.566	1.00		0.739												
**Monthly disposal income**																		
1^st^ Tertile	0.92	0.38 – 1.95	0.000			0.000												
2^nd^ Tertile	0.76	0.32 – 1.85	0.842	1.53	0.51 – 4.55	0.449												
3^rd^ Tertile	1.00	Reference	0.549	1.24	0.41 – 3.76	0.703												
**Occupation**																		
Unemployed	0.92	0.43 – 1.95	0.000	0.53		0.000												
Student	0.56	0.15 – 2.16	0.820	0.69	0.21 – 1.34	0.182												
Employed	1.00	Reference	0.401	1.00	0.18 – 2.71	0.692												
**Membership in a social group**																		
Yes	1.10	0.54 – 2.45	0.801	1.32	0.56 – 3.09	0.522												
No	1.00																	
**Religion**																		
Christian	1.51	0.32 – 7.13	0.602	2.02	0.25 – 16.34	0.512							1.79	0.37 – 8.77	0.471	1.68	0.20 – 4.28	0.63
Others	1.00			1.00									1.00			1.00		
**Parity**																		
One child	0.92	0.38 – 2.19	0.000	1.45	0.53 – 3.96	0.000							0.80	0.26 – 2.46	0.693	2.33	0.65 – 8.32	0.19
Two child	1.52	0.62 – 3.72	0.842	1.20	0.37 – 3.88	0.471							1.49	0.52 – 4.26	0.457	2.39	0.60 – 9.50	0.20
Three or more children	1.00		0.363	1.00		0.761							1.00			1.00		

## Discussion

This study was designed to describe and analyse socio-economic/demographic determinants and predictors to parental knowledge about immunisations which may be vital for a successful implementation process of the PCV-13. There was a generally high level of awareness regarding the perceptions and opinions of respondents about pneumonia disease burden and immunisations/EPI vaccines. However, only 19% of those sampled were aware of the availability of the PCV-13. In the multivariate adjusted models; parental educational level, income level, membership status in a social group and Region of origin were identified as important socio-economic/demographic determinants and predictors to parental knowledge of pneumonia disease burden, prevention and immunisations/EPI vaccines [19].

Similar observations have been reported earlier in Nigeria and Rajasthan-India [9, 10]. The higher levels of knowledge on the symptoms of pneumonia (69%) and on a preventive method (97%) found amongst parents/guardians which is similar to that reported in the study by Olumuyiwa & colleagues [9]. This may be attributed to the content of information given to them during antenatal visits. The fact that only 19% of the participants were aware of the availability of the PCV in our study stresses the need for an improvement in the quality of health information on pneumonia disease burden and prevention. However, this differs from the low rate (4%) of knowledge about Oral Polio Vaccine (OPV) reported in a Niger study [23], and the low rate of awareness (1%) that measles was vaccine-preventable in another study in Nigeria [24].

We observed that, the high and positive levels of awareness and knowledge of participants in our study could also be attributable to other socio-economic and demographic factors. For instant, over seventy-eight percent of the parents/guardians sampled had obtained educational levels above primary school. This too is an effect of free and compulsory primary education instituted in Cameroon since the early nineties. Adult literacy rate were reportedly high in a previous survey with 67% for females and 81% for males [25].

Similarly, as in our study, Olumuyiwa et al. [9] did not report any interference between vaccinations and the religious or cultural beliefs of the studied community in Nigeria although such controversies were a subject of contention in most parts of Nigeria [9]. Most parents/guardians did not think that to take their children to be vaccinated was a time-wasting exercise. Most often than not, parents stated that, “the health of a child is a major priority and has no price-tag since prevention was better than cure”. On the contrary, the issue of long-waiting time was mentioned as a potential risk factor on why children fail to get immunised in an Egyptian study and a recent study in Ibadan-Nigeria [8, 26].

Also, our findings show a high proportion of parents had a positive impression about the attitudes of health personnel at the vaccination units. Inversely, a Greek study reported that unfriendly health provider attitude and poor organisation at preventive services had contributed in an increase in unvaccinated children [27]. However, if this situation was not evident in our study, it does not cancel its existence and needs to be seriously considered. For it may likely be that, as some respondents stated, “The attitudes of the nurses at the vaccination units are also dependent on the institution on the one hand and the individual on the other”. Although this still needs further investigation, it is a popular belief that faith-based and private health services provide a better quality of care than some government managed ones [19].

The results again indicated that, 56% of the participants were of the opinion that an increase in public sensitisations and mass vaccination campaigns would be vital if the PCV-13 is to reach every child. Related findings had earlier been reported in an Indian study [10]. Although alternatives include increase in the level of health information, education and communication, and improvements in the social mobilisation/communication strategies; a synergy of these population-based opinions and alternatives could yield fascinating outcomes in attaining high vaccine coverage rates.

In the multivariate analyses, we documented a number of important associations between socio-economic determinants and predictors to parental knowledge about immunisations/EPI vaccines and the PCV-13 [19]. Firstly, parents/guardians in our study who were members in a social group (network) were more likely to have good knowledge on pneumonia disease burden. This complies with a Bangladesh study in which interpersonal communication was used among mothers in a social network resulting to significant improvement in vaccination uptake and coverage rate [28].

Secondly, it's no surprise that parents with lower education were found to have decreased odds to knowledge on the seriousness/ consequences of pneumonia; as confirmed in a related study [29]. Thirdly, parents in the lower income tertile and those having primary education or below were associated with reduced odds to good knowledge on the causes/risk factors of pneumonia infections. The relationship of education as a predictor to knowledge on immunisation-related aspects had earlier been emphasised [5, 9, 29–31]. While in a study on the determinants of the influenza vaccination in hard-to-reach urban populations in the United States, lower annual income was amongst the factors significantly associated with an interest of being vaccinated [32]. It is evident in relation to our study that parents/guardians in the lower income level may equally have an interest in the PCV-13 for their children but this maybe handicapped by their limited knowledge on the causes/risk factors and the dangers of pneumonia. As such, it will be essential to design a specific health information package on pneumonia disease burden and prevention for parents/guardians in the lower educational and income tertile levels.

Fourthly, our results revealed that, parents/guardians in the student occupational group were less likely to have good knowledge of the prevention against pneumonia. This is unexpected given that the student group, more educated than the average, is expected to be more knowledgeable on the matter. In related studies, parental occupation defined as the socio-economic condition of the household was shown to have statistically significant association with acceptance (and by implication, knowledge) of immunisation [33, 34]. This was in contrast to our finding. Previous studies had also shown that higher caretakers’ educational background was associated with increase knowledge and opportunity to get their children vaccinated [30, 35]. Although this is unclear, there may be a disparity when caretakers have a dual responsibility of child care, pursuing personal studies or working to sustain the household as it was peculiar in our study community.

Lastly, we observed an association between parental Region of origin and their knowledge on the availability of vaccines against pneumonia infections. Parents from the Adamawa, North and Extreme North Regions were likely to be more aware than those from the other regions. In two separate studies in Bangladesh and Ghana, similar associations between parental regions of residence and their knowledge and acceptance of vaccination were reported [34, 36]. Although this may be aligned to many factors, a striking observation in our study is the concept of social networking (parental membership in a social group). We may have little feasible explanation except that people from these three Northern Regions (residing within or out of their communities) are believed to establish and propagate strong social network groups in which developmental issues of their localities and other matters are usually discussed on a regular basis. Hence, it is not uncommon to share information on health especially with regards to immunisations.

When considering the validity of the present findings, certain aspects are to be equally taken into account. Of all those contacted, over 73% participated in the study, from which 82% were included in the final analysis. Hence, given the challenges in generating raw data from low-resource settings, the original material presents well a reflection of the total population in the area. However, although a high response rate may eliminate the constraints associated with poor representativeness, the study does not minimise contributions which should had come from those who declined to participate or were excluded. It is not entirely understood why some of those contacted declined to participate in the study. The reason some people declined to participate in the parental sample may be the absence of a well-informed parent/guardian at the household at the time of the study. The rest of those excluded were on grounds of not meeting all of the inclusion criteria.

This study was geographically limited to two health districts (out of 179), in a semi-urban slum due to financial constraints. Hence, the results may be specific to the characteristics of these zones. However, the choice by convenience of these two health districts for the study in a semi-urban cosmopolitan zone is also vital considering that participants originated (by chance) from many Regions. Thus, it is unlikely that any differences between those who were excluded or declined to participate and the included participants will result to any major bias.

Furthermore, the fact that the findings from the study area were comparable with those from related studies suggests the results might also apply or be obtained elsewhere under similar conditions. But few other data are available from developing countries on new vaccines introduction to confirm or reject this assumption [19], hence, a need for profound research.

## Conclusion

Thus, the introduction of the PCV-13 into the national Expanded Programme on Immunisation of Cameroon is a welcome agenda. Although a majority of parental opinion weighs on increasing public sensitisation if the PCV-13 is to reach every child, other issues need to be borne in mind. The health system, political will and other structural barriers could not be independent to give PCV-13 a wider access.
